# Rekonstruktion großer osteochondraler Defekte des distalen Femurs und der proximalen Tibia: Anpassung frisch gefrorener Allografts unter Zuhilfenahme 3D-gedruckter Kunststoffmodelle

**DOI:** 10.1007/s00113-020-00846-y

**Published:** 2020-08-10

**Authors:** E. Fleischhacker, D. Ehrl, J. Fürmetz, R. Meller, W. Böcker, C. Zeckey

**Affiliations:** 1 Klinik für Allgemeine, Unfall- und Wiederherstellungschirurgie, LMU Klinikum, Ludwig-Maximilians-Universität München, Marchioninistr. 15, 81377 München, Deutschland; 2 Abteilung für Hand-, plastische und ästhetische Chirurgie am LMU Klinikum, Ludwig-Maximilians-Universität, 81377 München, Deutschland; 3 Klinik für Orthopädie und Unfallchirurgie, Klinikum Dritter Orden München, 80638 München, Deutschland; 4 Klinik für Unfallchirurgie und Orthopädie, RoMed Klinikum Rosenheim, 83022 Rosenheim, Deutschland

**Keywords:** Defektrekonstruktion, Frisch gefrorene Allografts, 3D-gedruckte Modelle, Osteochondrale Rekonstruktion, Dreidimensionaler Druck, Reconstruction of defect, Fresh frozen allograft, 3D-printed model, Osteochondral reconstruction, Three dimensional printing

## Abstract

Die Rekonstruktion großer osteochondraler Defekte stellt nach wie vor eine Herausforderung in der muskuloskeletalen Chirurgie dar. Frisch gefrorene Allografts sind eine häufig genutzte Ressource für die Behandlung solcher Gewebedefekte. Darüber hinaus ermöglichen 3D-gedruckte Kunststoffmodelle vielfältige Optionen in der präoperativen Planung und bei der intraoperativen Anpassung der Transplantate, sodass sie optimal einheilen und das bestmögliche funktionelle Ergebnis für den Patienten erreicht wird.

## Abstract (en)

The reconstruction of large osteochondral defects is still a challenge in musculoskeletal surgery. Fresh frozen allografts are a frequently used resource for the treatment of such tissue defects. Furthermore, 3D-printed models enable multiple options in the preoperative planning and intraoperative adaptation of the allografts, so that healing is optimal and the best functional outcome for the patient is achieved.

## Hintergrund

Offene Frakturen des Kniegelenks mit signifikantem Verlust an osteochondraler Substanz kommen bei jungen Patienten vergleichsweise selten vor, haben jedoch meist fatale Konsequenzen. Aufgrund des hohen Aktivitätslevels und des funktionellen Anspruchs stellt die Behandlung solcher Fälle den Chirurgen vor große Herausforderungen. Obwohl verschiedene Therapieoptionen in der Literatur beschrieben werden, herrscht hier kein Konsensus.

Um die Patienten vor einem Extremitätenverlust zu bewahren, bieten Allografts eine Möglichkeit, osteochondrale Defekte zu rekonstruieren und so die Extremität zu erhalten [15]. Die Vorteile dieses Verfahrens liegen in der unbegrenzten Größe des knöchernen sowie knorpeligen Defekts und damit in der Möglichkeit, auch große subchondrale Knochenverluste zu rekonstruieren [15]. Während Verfahren, welche überwiegend bei großen Knorpeldefekten indiziert sind, wie die „fresh large osteochondral allograft transplantation“ (FLOCSAT), den subchondralen Knochen nur als Trägermatrix nutzen [7], kann mithilfe von Allografts auch ein substanzieller Knochendefekt rekonstruiert werden. Des Weiteren enthalten Allografts reifen hyalinen Knorpel und keinen Faserknorpel [2, 7]. Bei weitgehend intaktem Knochen kann nach sicherer Sanierung eines möglichen Infekts auch die Primärimplantation einer Endoprothese in Erwägung gezogen werden. Hierzu muss allerdings eine gute Knochengrundlage vorhanden sein, um die Prothese sicher zu verankern. Des Weiteren können auch hier häufig keine Standardimplantate, sondern nur Sonderanfertigungen verwendet werden [14].

Derzeit sind 3 verschiedene osteochondrale Allograft-Typen auf dem Markt [3]. Frische Allografts werden nach der Entnahme nativ zwischen 4 und 37 °C aufbewahrt, hierdurch ist ihre Haltbarkeit auf 12 bis 14 Tage beschränkt, jedoch haben sie den höchsten Anteil vitaler Zellen [3, 16]. Um kryokonservierte Allografts bei −80 °C einfrieren zu können, müssen sie in Frostschutzmitteln, wie Glycerol oder Dimethylsulfoxid (DMSO), gelagert werden, was zu einem höheren Zelluntergang führt und so das Outcome nach Implantation reduziert, die Haltbarkeit der Transplantate jedoch deutlich verlängert [13]. Frisch gefrorene Transplantate hingegen haben eine begrenzte Haltbarkeit, weil sie nicht in einem Frostschutzmittel (Glycerol oder DMSO) und bei höheren Temperaturen als kryokonservierte Allografts gelagert werden. Somit haben frisch gefrorene Allografts aber auch einen höheren Anteil vitaler Chondrozyten. Aufgrund der genannten Nachteile frischer und kryokonservierter Transplantate stellen frisch gefrorene Allografts die beste Option zur Rekonstruktion großer osteochondraler Defekte dar [8, 13, 15]. Nichtsdestotrotz sind diese Transplantate in ihrer Anzahl und Verfügbarkeit begrenzt, was auch an der aufwendigen und kostenintensiven Transplantatgewinnung und -lagerung liegt [7]. Hinzukommt, dass das Augenmerk der Öffentlichkeitsarbeit v. a. auf der Organspende liegt, jedoch nur wenig über eine Gewebespende informiert wird. Dies hat zur Folge, dass die Gewebespendebereitschaft in Deutschland über die vergangenen Jahre weitgehend konstant geblieben ist und keinen nennenswerten Zuwachs erfahren hat, sodass diese Ressourcen weiter knapp sind [1]. Die genannten Limitationen führen dazu, dass den rekonstruierenden Chirurgen häufig nur zu große oder zu kleine Allografts zur Verfügung stehen, welche dann auf den Defekt des jeweiligen Patienten konfektioniert werden müssen.

Die Anpassung der Allografts stellt daher eine wesentliche Herausforderung für den Chirurgen dar. Vereinfachen kann man dies durch die Zuhilfenahme dreidimensionaler (3D) gedruckter Kunststoffmodelle, welche auf präoperativen Planungs-CTs beruhen [11]. Die präoperativ gedruckten Modelle der Knochen und Defekte des Patienten helfen dem Chirurgen, die Pathologie in ihrer Dreidimensionalität zu begreifen und so die Anpassung der Transplantate präoperativ präzise zu planen und intraoperativ zu steuern [5, 11].

Dennoch konnte nach eingehender Literaturrecherche keine Fallbeschreibung gefunden werden, in der ein substanzieller osteochondraler Defekt von Tibia und Femur unter Zuhilfenahme 3D-gedruckter Kunststoffmodelle mit frisch gefrorenen Allografts rekonstruiert wurde.

## Fallbericht

**Figure 1:**
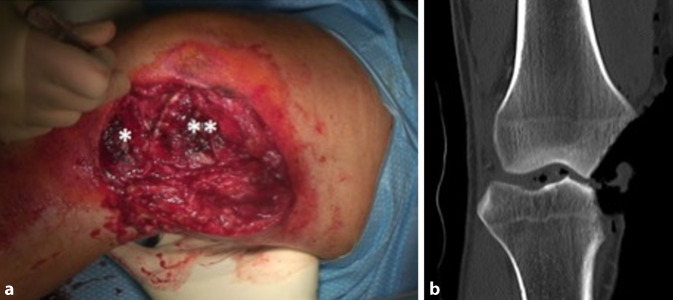

Ein 56 Jahre alter männlicher Patient, mit nur leichtem Hypertonus in der Vorgeschichte, wurde als Fahrradfahrer in einen Verkehrsunfall verwickelt, bei dem er von einem wendenden Lkw erfasst und einige Meter über den Asphalt geschliffen wurde. In der initialen klinischen Untersuchung zeigte sich ein ca. 10 cm durchmessender Defekt am medialen rechten Knie. Er entsprach einer offenen Fraktur Grad IIIb (nach Gustilo-Anderson [4]) der medialen Femurkondyle sowie des medialen Tibiaplateaus (Abb. 1a). Des Weiteren lagen eine Ruptur des Innenmeniskus sowie aller Anteile des medialen Kollateralbandapparats vor. Im CT zeigten sich ein Defekt der medialen Femurkondyle mit einem Volumen von 14,3 cm^3^ sowie ein osteochondraler Defekt am medialen Tibiaplateau mit einem Volumen von 5,3 cm^3^ (Abb. 1b).

Im einem ersten Eingriff wurde die Wunde débridiert und der Hautdefekt mit EpiGARD© (Biovision GmbH, Ilmenau, Deutschland) verschlossen. Zur Ruhigstellung erhielt der Patient eine dorsale Gipsschiene. Am Tag nach dem Unfall wurde er dann im Rahmen des Traumanetzwerks in das überregionale Traumazentrum verlegt. Nach mehrfachen Operationen u. a. mit Wunddébridement und Anlage bzw. Wechsel von Vakuumverbänden wurde im Verlauf, zusammen mit den Kollegen der plastischen Chirurgie, die Rekonstruktion des Defekts geplant.

**Figure 2:**
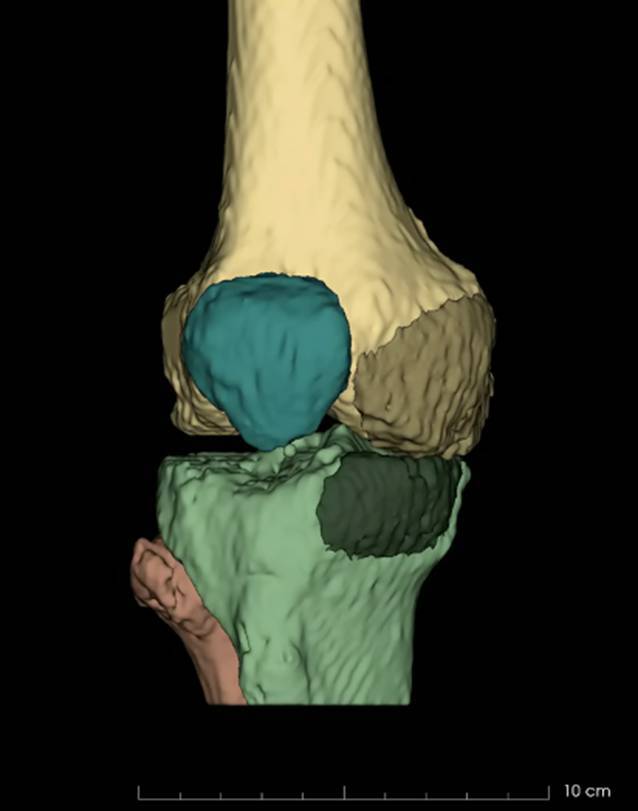

Hierfür wurde eine CT beider Kniegelenke mit einer Schichtdicke von 1 mm angefertigt. Anschließend wurde das unverletzte linke Knie mithilfe des „Open-source“-Programms „3D slicer“ virtuell gespiegelt. Nachdem die Modelle der beiden Knie übereinandergelegt worden waren, ließen sich die Defekte an Tibia und Femur rekonstruieren, ausmessen und das Volumen berechnen (Abb. 2). Anhand der Referenzierung des kontralateralen Femurs sowie der kontralateralen Tibia konnten im Anschluss vergleichbare Allografts selektiert werden. Daraufhin erfolgte die terminierte Order der niederländischen Transplantate. Um die Defekte und dazugehörigen osteochondralen Fragmente für den Operateur begreiflich zu machen, wurden sie außerdem mithilfe eines 3D-Druckers (Sigmax; Fa. BCN3D) aus Polylactid(PLA)-Kunststoff gedruckt. So war es den Chirurgen möglich, die Defekte detailliert zu studieren, um schließlich die Rekonstruktion zu planen.

**Figure 3:**
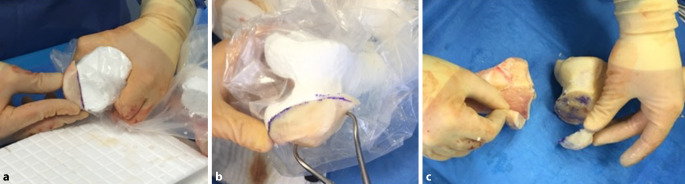

**Figure 4:**
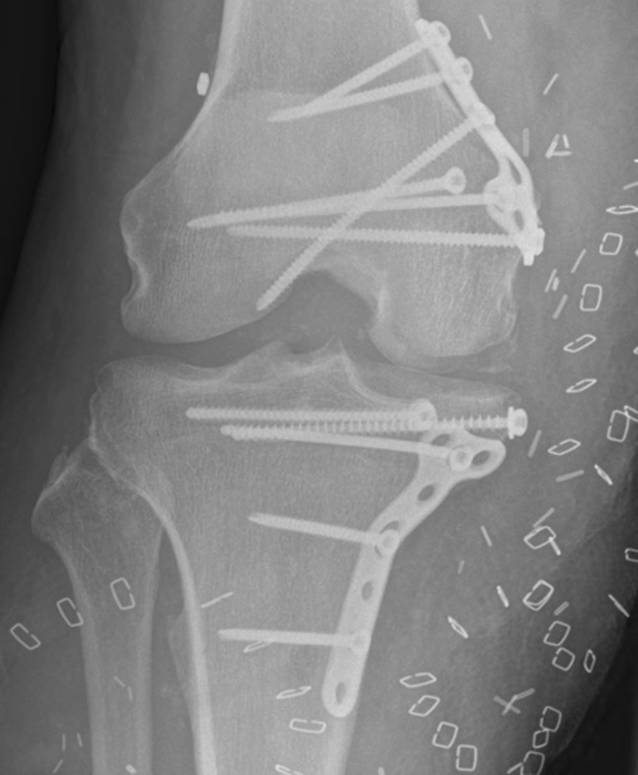

Diese Operation wurde 44 Tage nach dem Unfall in Rechtsseitenlage durchgeführt; so konnten alle Operationsschritte vorgenommen werden, ohne den Patienten umzulagern. Für die Rekonstruktion des medialen Bandapparats wurden zunächst die Sehnen des M. semitendinosus und des M. gracilis am linken Knie entnommen. Anschließend wurde der Innenmeniskus offen mit einem monofilen, resorbierbaren Faden (Maxon^TM^; Fa. Medtronic) der Stärke 2‑0 refixiert. Dann erfolgte die Präparation der knöchernen Defekte. Hierzu wurden die Umrisse der osteochondralen Defekte zunächst anhand der steril verpackten Modelle auf das tibiale und das femorale Allograft übertragen und schließlich die zu ersetzenden Fragmente in Form gebracht (Abb. 3a, b). Während dieses Vorgangs konnte der Operateur durch Anlegen und Vergleichen mit dem Modell eine präzise Kopie des osteochondralen Defekts erstellen (Abb. 3c). Um eine gute Osseointegration der Allografts zu gewährleisten, wurden die Tibia und das Femur des Patienten mikrofrakturiert. Danach erfolgte die Fixierung der Allografts mit nichtwinkelstabilen Platten und Schrauben. Für das femorale Allograft wurden eine T‑förmige 4‑Loch-Kompressionsplatte sowie eine 3,5 mm durchmessende Zugschraube mit Vollgewinde verwendet. Das tibiale Allograft wurde mit einer T‑förmigen 5‑Loch-Abstützplatte sowie zwei 3,5 mm durchmessenden Zugschrauben mit Vollgewinde und Unterlegscheiben fixiert. Die Rekonstruktion des medialen Bandapparats erfolgte dann anatomisch mit oben genannten autologen Sehnentransplantaten in „Double-bundle“-Technik, wie von Petersen et al. 2017 beschrieben [12]. Hierzu wurden zunächst Zieldrähte in Tibia und Femur platziert und anschließend überbohrt. Aufgrund der Konfiguration der Defekte war es nötig, das Bohrloch im Condylus medialis femoris und das Bohrloch für die anatomische tibiale Verankerung des hinteren Schrägbandersatzes (POL) durch die Transplantate zu setzen. Die augmentierten und angeschlungenen Sehnen wurden durch das tibiale Bohrloch mithilfe eines knotenlosen TightRope-Systems (TightRope®, Fa. Arthrex) gezogen. Der weichteilige Defekt wurde anschließend mit einem myokutanen Latissimus-dorsi-Lappen und Spalthaut verschlossen. Der intraoperative Bewegungsumfang des rechten Kniegelenks betrug Extension/Flexion 0°/0°/120°, und das Knie war in allen Dimensionen stabil. Die postoperativen Röntgenbilder sind in Abb. 4 dargestellt.

In den ersten 6 Wochen wurde der Bewegungsumfang des Knies mit einer Orthese auf 60° Flexion, bei freier Extension, limitiert. Des Weiteren wurde der Patient an Unterarmgehstützen mit Bodenkontakt mobilisiert. Die Lappenplastik heilte regelrecht ohne Wundheilungsstörung. Bis auf eine Thrombose der ipsilateralen V. poplitea, welche keine weiteren Folgen hatte, traten keine Komplikationen auf.

**Figure 5:**
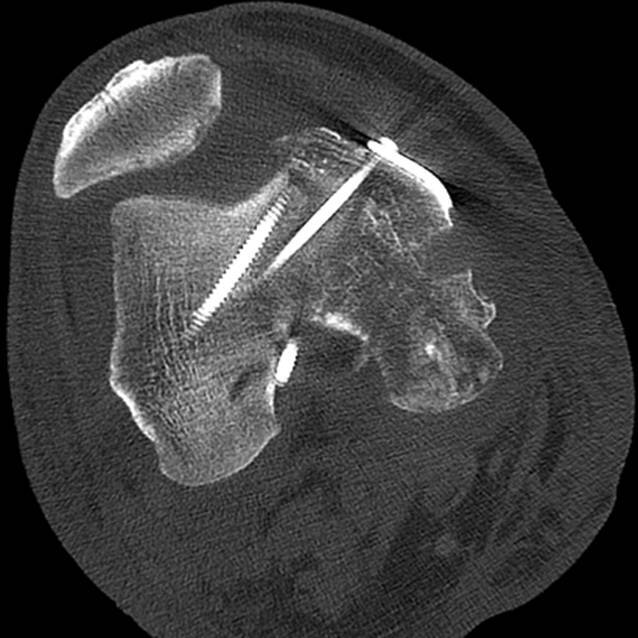

In der CT, 5 Wochen postoperativ, zeigte sich eine Konturunregelmäßigkeit am femoralen Allograft (Abb. 5), welche kurz darauf arthroskopisch und komplikationslos entfernt werden konnte. Intraoperativ zeigten sich die Allografts vital. Danach begann der Patient, an 2 Unterarmgehstützen, bei freiem Bewegungsumfang, aufzubelasten. Der Heilungsverlauf der Rekonstruktion des medialen Kollateralbandapparats verlief ebenfalls regelrecht. In der klinischen Untersuchung zeigte sich im Seitenvergleich, weder in Streckung noch in 30°-Beugung, eine vermehrte mediale oder laterale Aufklappbarkeit. Ebenso zeigten sich in der klinischen Untersuchung keine pathologische Auffälligkeit in der klinischen Meniskustestung und kein Hinweis auf eine Abweichung der Beinachse.

**Figure 6:**
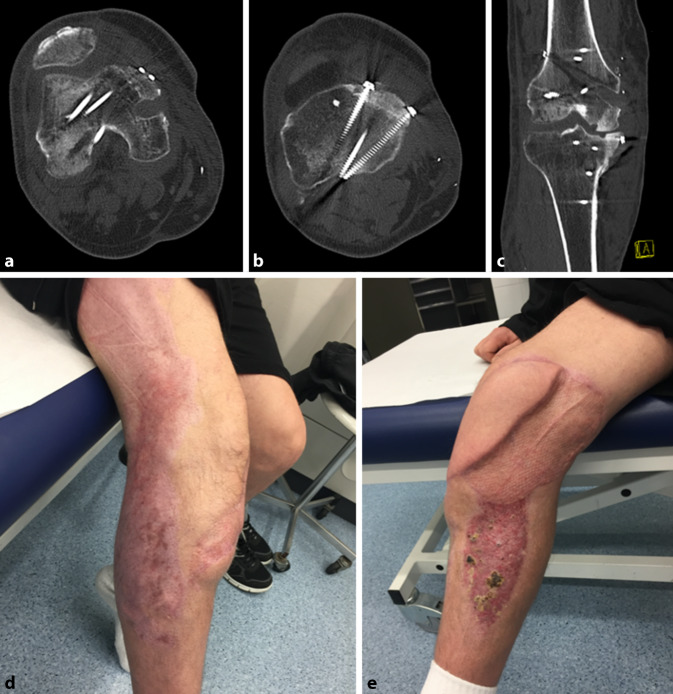

Fünf Monate nach der Rekonstruktion war der Patient ohne Hilfsmittel mobil. Im CT zeigte sich ein zufriedenstellendes Ergebnis ohne Hinweis auf einen Untergang der Transplantate (Abb. 6a, b). Allerdings waren im Bereich der chondralen Übergänge kleine umschriebene Osteolysen (Abb. 6c) zu sehen. Insgesamt sind außerdem radiologische Zeichen einer Inaktivitätsosteopenie, im Sinne fleckiger Entkalkung, abzugrenzen. Dennoch hatte der Patient keine Schmerzen im rechten Knie, konnte Spaziergänge von 2 h problemlos bewältigen und verneinte eine regelmäßige Analgetikaeinnahme. Er beklagte lediglich noch eine Flexionseinschränkung von etwa 30°. Auch die weichteilige Rekonstruktion der plastischen Chirurgen heilte bis zu diesem Zeitpunkt regulär (Abb. 6d, e).

## Diskussion

Die Rekonstruktion substanzieller osteochondraler Defekte des Kniegelenks stellt für Chirurgen noch immer eine große Herausforderung dar. Obwohl in der Literatur verschiedene Vorgehensweisen, v. a. für kleine Läsionen, beschrieben wurden, wird die Rekonstruktion großer Defekte weiter kontrovers diskutiert. In diesem Fallbericht wird, im kurzfristigen Verlauf, ein gutes Outcome durch die Verwendung frisch gefrorener Allografts beschrieben, welche, unter Zuhilfenahme von 3D-Modellen, auf die Defekte angepasst werden konnten.

Die meisten Publikationen über Rekonstruktionen großer osteochondraler Defekte des Knies beschreiben eine Behandlung nach Tumorresektion, wohingegen wenig über die Rekonstruktion traumatisch erworbener Defekte veröffentlicht wurde. Fagan und Furey publizierten einen Fallbericht über einen 19-jährigen männlichen Patienten, der nach einem Unfall einen osteochondralen Defekt der medialen Femurkondyle aufwies [2]. Auch bei diesem Patienten wurde ein frisch gefrorenes Allograft verwendet, was 2 Jahre nach Rekonstruktion ein gutes klinisches Outcome aufwies. Allerdings wird in diesem Bericht nichts über die präoperative Planung und intraoperative Anpassung des Transplantats berichtet. Der Sitz und die Bündigkeit entscheiden jedoch über die Qualität der Osseointegration und damit über die Funktion des Gelenks [15]. Im Jahr 2004 untersuchten Koh et al. in einer biomechanischen Studie den Zusammenhang zwischen Transplantathöhen-Mismatch und Kontaktdruck. Sie zeigten, dass bereits 0,5–1 mm Hochstand 50 % mehr Kontaktdruck nach sich ziehen und so eine frühzeitige Destruktion der Chondrozyten resultiert [6]. Aufgrund dieser Erkenntnisse wird umso deutlicher, wie wichtig es ist, das Transplantat möglichst anatomisch auszugestalten.

Durch virtuelle Spiegelung des unverletzten Knies können die osteochondralen Defekte in Größe und Form rekonstruiert werden. So lässt sich eine größtmögliche Ähnlichkeit zur ursprünglichen ossären Struktur erreichen. Dieses Prinzip wurde bereits 2011 von Liacouras et al. beschrieben, die so eine aurikuläre Prothese herstellten [9]. Sie benutzen ebenfalls Bilder der gesunden Gegenseite und spiegelten diese, um die Prothese möglichst anatomisch zu gestalten [9].

Aufgrund der Größe und Komplexität der Defekte im vorgestellten Fall war es essenziell, intraoperativ beim Anpassen der Grafts eine Referenz zu haben [10]. Durch die Kunststoffmodelle bekommt der Chirurg neben der virtuellen 3D-Rekonstruktion auch ein taktiles Feedback, welches ihm hilft, den osteochondralen Defekt zu verstehen und das Transplantat anzupassen [10]. 3D-gedruckte Modelle werden bereits regelhaft bei der Rekonstruktion und Osteosynthese von Acetabulumfrakturen sowie bei der Rekonstruktion kraniofazialer Defekte verwendet [5, 10]. Dabei wird diese Methode v. a. zum Anbiegen von Osteosyntheseplatten und anderen Implantaten verwendet, aber auch zur Rekonstruktion größerer knöcherner Defekte wie im oben beschriebenen Fall.

## Fazit für die Praxis

- Frisch gefrorene Allografts sind eine gute Option zur Behandlung großer osteochondraler Defekte.
- Eine optimale anatomische Anpassung der Transplantate ist essenziell.
- 3D-gedruckte Kunststoffmodelle eröffnen vielfältige Möglichkeiten in der präoperativen Planung.
- Durch die Verwendung 3D-gedruckter Kunststoffmodelle kann der Chirurg die Transplantate präzise anpassen und schließlich ein gutes funktionelles Ergebnis erreichen.
